# A Spatial Analysis of Health Disparities Associated with Antibiotic Resistant Infections in Children Living in Atlanta (2002–2010)

**DOI:** 10.5334/egems.308

**Published:** 2019-09-12

**Authors:** Fatima Ali, Lilly C. Immergluck, Traci Leong, Lance Waller, Khusdeep Malhotra, Robert C. Jerris, Mike Edelson, George S. Rust

**Affiliations:** 1 Louisville Metro Department of Public Health and Wellness, US; 2 Morehouse School of Medicine, US; 3 Emory University, Rollins School of Public Health, US; 4 Temple University, US; 5 Emory University, School of Medicine, US; 6 InterDev, US; 7 Florida State University, College of Medicine, US

**Keywords:** antibiotic resistant bacteria, community-associated MRSA, pediatrics, spatial analyses

## Abstract

**Background:**

Antibiotic resistant bacteria like community-onset methicillin resistant *Staphylococcus aureus* (CO-MRSA) have continued to cause infections in children at alarming rates and are associated with health disparities. Geospatial analyses of individual and area level data can enhance disease surveillance and identify socio-demographic and geographic indicators to explain CO-MRSA disease transmission patterns and risks.

**Methods:**

A case control epidemiology approach was undertaken to compare children with CO-MRSA to a noninfectious condition (unintentional traumatic brain injury (uTBI)). In order to better understand the impact of place based risks in developing these types of infections, data from electronic health records (EHR) were obtained from CO-MRSA cases and compared to EHR data from controls (uTBI). US Census data was used to determine area level data. Multi-level statistical models were performed using risk factors determined *a priori* and geospatial analyses were conducted and mapped.

**Results:**

From 2002–2010, 4,613 with CO-MRSA and 34,758 with uTBI were seen from two pediatric hospitals in Atlanta, Georgia. Hispanic children had reduced odds of infection; females and public health insurance were more likely to have CO-MRSA. Spatial analyses indicate significant ‘hot spots’ for CO-MRSA and the overall spatial cluster locations, differed between CO-MRSA cases and uTBI controls.

**Conclusions:**

Differences exist in race, age, and type of health insurance between CO-MRSA cases compared to noninfectious control group. Geographic clustering of cases is distinct from controls, suggesting placed based factors impact risk for CO-MRSA infection.

## Introduction

Ever since John Snow identified the Broad Street public water pump as the source of cholera in London in 1854, the cluster-mapping of infectious diseases has been an important tool for understanding the spread of disease and identifying potential public health interventions. Antibiotic resistant bacterial infections, such as community-onset methicillin resistant *Staphylococcus aureus* (CO-MRSA) infections have continued to cause significant infections in both inpatient and outpatient settings since they were first observed in the mid-1990s [1,2,3]. The number of children hospitalized with MRSA infections increased from 6.7 cases per 1,000 admissions in 2002 to 21.2 cases in 2007 [4]. Population based studies have shown a higher rate of invasive MRSA infections among blacks and other minorities [5,6,7,8]. Community-onset MRSA infections have been endemic in many urban areas with significantly increased risk for invasive infection among blacks [7,9,10]. It has been suggested that this disparity in risk for invasive disease may be due to socioeconomic factors [11,12], but this is not well studied for CO-MRSA infections or how it contributes to disease patterns, particularly among children [6,7,9]. Socio-environmental and ecological factors, reflecting communities in which patients reside, have been shown to be important risk factors of CO-MRSA colonization and infection in children [13,14]. For example, household crowding has been cited as a risk for MRSA infections, but the impact of neighborhood crowding has not previously been addressed [15,16].

Unintentional traumatic brain injury in children less than 15 years of age has not been associated with health disparities [17], even though there are disparities in the treatment rendered to children who have sustained traumatic head injuries. Among US children aged 0 to 17 years, the lifetime estimate of a parent-reported unintentional traumatic brain injury was 2.5 percent, which represents 1.8 million US children with increased risk among non-Hispanic white, boys, and those with private health insurance [18]. In 2013, there were approximately 640,000 traumatic brain injury related emergency department visits among children less than 14 years of age [19]. Children under 4 years had the highest rates of unintentional brain injury ED related visits across all age groups of children and adults. (In 2012, sports and recreational activity, accounting for 325, 000 ED visits for unintentional head injury among children and adolescents, also were major causes for unintentional brain injury) [17]. We hypothesized that (1) socio ecological risks would differ between cases (CO-MRSA) and controls (unintentional traumatic brain injury), and (2) spatial diffusion model of spread seen with mapping of infectious conditions [20,21] such as CO-MRSA infections, would not be seen in children with unintentional TBI (uTBI).

The use of geographic information systems (GIS) in public health allows for the identification of disease clusters and a better understanding of the relationship between spatial patterns and spread of disease [22]. It remains a useful tool in epidemiologic research to explore environmental and social factors, which may impact health outcomes [23,24,25]. While most studies tend to focus exclusively on individual level risk factors, understanding population-level factors that incorporate place based socioeconomic and environmental factors may help discern what influences transmission and therefore, develop strategies to prevent further spread [26]. This is the first large scale study to compare, using multilevel spatial analyses, placed based socio ecological risk factors between antibiotic resistant bacterial infections and a non infectious condition of (uTBI) among children living in an urban setting.

## Methods

### Study Design

A case-control epidemiology study was conducted on patients treated from two major pediatric hospitals located in Atlanta, Georgia from 2002 through 2010. Patients who were treated for a CO-MRSA infection in the emergency department or inpatient were identified as cases. These case patients were included only if they met the definition of CO-MRSA: (1) Patients had clinical condition compatible with a *S. aureus* infection, according to the International Classification of Disease Clinical Modification (ICD-CM 9) and a positive *S. aureus* culture from the site of infection with evidence of resistance to the antibiotic, oxacillin. (2) Community-onset infections were defined as those which occurred within or at 48 hours of hospital admission for inpatients and all outpatients [27,28]. (Cultures obtained after 48 hours of admission to the hospital were classified as ‘hospital or healthcare onset’ and were excluded from this study).

All patients who entered the same hospitals as cases, with a diagnosis of unintentional traumatic brain injury (uTBI) based on ICD-CM 9 codes were used as controls. In order to explore place based socio ecological factors that are driven by a spatial diffusion model, we chose to compare our infectious condition (CO-MRSA) to the non-infectious condition of uTBI [29].

A 20-county Metropolitan Statistical Area (MSA) was used as a geographic boundary to represent the catchment area of the pediatric hospitals. This area was chosen due to its use by the Georgia Emerging Infections Active Bacterial Core surveillance program (antibiotic resistant bacteria, MRSA, is one of the bacterial infections monitored in this state and is a federally funded program) [30]. Furthermore, Children’s Healthcare of Atlanta three free standing pediatric hospitals and outpatient centers capture ~88.6 percent of the pediatric hospital discharges for this 20-county MSA [31]. We included the two largest hospitals of this pediatric health care system, which represents patients seen at its tertiary and quaternary care centers. (The third hospital was not included for this study due to incomplete data for the variables of interest.) Since our study data included a 9-year period and two national census reference data (US Census 2000 and US Census 2010), we chose to calculate community or area variables for patients enrolled from 2002 through 2005 using US Census 2000 and area variables from 2006 through 2010 using US Census 2010. Based from US Census 2000, there were 1,894 block groups [32], and from US Census 2010, there are 2,492 block groups in this 20-county MSA [33]. Only resident addresses that fell within this designated geographic boundary were included in the analyses. Addresses that were out-of-state, invalid, including post office box numbers and ungeocodable addresses, were excluded.

### Patient Level Descriptions

From electronic health records (EHR), demographic information (age of patient at the time of hospital admission, race and ethnicity, gender, and type of health insurance) were recorded for each patient. Race was categorized as white, black, and other (Asian, Native American, Multiracial, Native Hawaiian, and other/declined). Ethnicity was stratified as Hispanic or non Hispanic. Age was the only continuous variable used in the analysis. Health insurance was categorized into public, private, or uninsured. Clinical data included source of MRSA culture, antibiogram of MRSA, clinical diagnosis at the time of the culture, past documentation from medical records of MRSA infections and other conditions.

### Neighborhood Level Descriptions

The US Census block group where a patient resided at the time of the hospital visit was identified for each case and control patient, and data to determine household crowding, proportion of black by block group, and proportion population below poverty were obtained. All block group data were obtained from either the US Census 2000 [32,34] or 2010 Short Form [33] or the 2009–2013 American Community Survey [35]. Boundary files for use in GIS for Georgia block groups and counties were downloaded from the National Historical Geographic Information System [36].

#### Household crowding

From the US Housing and Urban Development definition, household crowding was defined as any housing unit with more than one occupant per room [37]. Proportions of patients living in block groups with crowded households were determined.

#### Race and ethnicity

Race and ethnicity was also another variable of interest as risk for CO-MRSA are said to differ among race and ethnic groups with higher rates among blacks and lower among Hispanic/Latino populations [38,39]. Race and ethnicity proportions within each block group were determined from US Census 2000 for patients enrolled from 2002–2005, and from US Census 2010 for block groups identified from patients enrolled from 2006–2010. For this area variable, we divided into four categories: (1) White (Non-Hispanic); (2) Black (Non-Hispanic); (3) Hispanic; and (4) Other (Asian, Native American, Multiracial, Native Hawaiian, and other/declined). We assigned ‘concentrated’ area of blacks by using the overall percentage of blacks for the state as a reference point [40]. A block group was ‘black concentrated’ if the black population was more than the average percentage of black population for the state of Georgia. For example, the overall percentage of blacks in Georgia was 28.7 percent in 2000 and 30.5 percent in 2010, so any block group ≥28.7 percent blacks for patients enrolled from 2002–2005 or ≥30.5 percent blacks for patients enrolled from 2006–2010, was thereby defined as a ‘black concentrated’ area.

#### Poverty

The US Census Bureau’s poverty-to-income ratio was used to assess poverty status [41]. This measure is calculated by a family’s income before taxes, divided by poverty thresholds, which vary by family size. A ratio above 1 indicates that a family is above the poverty level. The poverty-to-income variable was categorized as below poverty if the ratio was less than 1. We then determined the proportion of a block group, which fell below poverty using this definition.

### Data Analysis

Chi-square tests were used to examine the distribution and relationship between CO-MRSA infections and unintentional traumatic brain injuries among categorical variables. Independent samples t-test was used to compare means of age between the two groups. Logistic regression was used to evaluate the relationship between individual and neighborhood level predictors (household crowding, socioeconomic status of households at the block group) and the dichotomous outcome, CO-MRSA versus unintentional TBI. Due to multiple encounters per person, we applied a generalized estimating equation (GEE) model using an exchangeable covariance structure with model based standard errors to adjust for the within patient correlation. Crude odds ratios (OR) were based on conditions determined *a priori* to be associated with risk of MRSA and used as estimates of relative risks. Adjusted odds ratios (aORs) were calculated using a generalized linear mixed model, allowing for random effects and correlated errors for non-normal data. Three separate models were applied for multiple regression analyses: Model 1–this model estimates the association between individual-level factors and CO-MRSA or uTBI by using a backward elimination logistic regression with a threshold p-value of 0.05 to remain in the model in order to obtain a parsimonious model [42]. Model 2–this model is similar to Model 1 except that estimates of association were made between neighborhood-level factors and CO-MRSA or uTBI. Model 3–for this model, a multilevel approach was used to estimate odds for both individual level and neighborhood level factors [42]. All individual (race/ethnicity, health insurance, gender, and age) and area (crowding, black concentrated) variables were treated as categorical. The only exception is poverty, for which we used percent below poverty as a continuous variable. All tests for significance were two-tailed, and a p-value of ≤0.05 was considered significant. Statistical analysis was performed using SAS version 9.4 (SAS Institute, Cary, NC).

### Spatial Analyses

Residential addresses from electronic health records for all CO-MRSA cases and uTBI controls identified for 2002–2010, were geocoded and thematically mapped, using ArcGIS version 10.3 for Desktop (ESRI, Redlands, CA). A neighborhood boundary for each geocoded address was then determined. Block groups (approximately 400 households or 1,200 individuals) were chosen to reflect neighborhood level information, since this is one of smallest geographic boundaries that can be linked with aggregated data from the US Census. Cumulative age-specific incidence rates were calculated for each block group using the total population of 0–18-years. Population data were retrieved from the 2000 and 2010 Census Summary File 1 [43]. To calculate density, we used the *ArcGIS Spatial Analyst Kernel Density* tool. The output is a surface raster layer depicting the density of cases. This was performed in series for each year of CO-MRSA and uTBI. Output density raster is classified into four classes using the *Jenks Natural Breaks* classification method. The lowest density of the four classes is not shown on the map (many of these areas have minimal cases). The remaining three classes are rendered on the map series as ‘High,’ ‘Medium,’ and ‘Low’ density areas.

Using Getis-Ord Gi* statistic, a hot spot analysis was performed using calculated incidence rates and non-aggregated data. Optimized hot spot analysis is a tool used to evaluate statistically significant spatial clustering of high and low values. Hot spots were based on cumulative incidence rates of CO-MRSA infections and uTBI for each block group in the 20-county MSA. To begin exploring the space and time relationship among CO-MRSA, we applied Space Time Cube tool and Emerging Hot Spot Analysis tool. In so doing, we aggregated the points (cases) into space-time ‘bins’ and within each bin, the cases were counted and the trend for ‘bin’ values across time (e.g., 2002–2004, Figure 2), at each location, was measured using the Mann Kendall trend statistic test, which carried out a rank correlation analyses. This space-time comparison was done for CO-MRSA, comparing 2002 to each successive year through 2010. (Supplemental data). For this analysis, we used the time step interval of a single year, and a default distance interval. In this manner, we were able to identify trends, which occurred over space and time by finding new, intensifying, diminishing and sporadic hot and cold spots within the 20 counties.

## Results

There were a total 4,613 cases of CO-MRSA and 34,758 controls of uTBI included in the analyses. The average ages were 5.27 years and 5.03 years for cases and controls, respectively. Whites made up the largest percentage of patients with uTBI (54 percent), while blacks (45 percent) and whites (43 percent) made up a similar percentage of patients with CO-MRSA infections; the distribution of race and ethnicity between CO-MRSA and uTBI was statistically significant (p < 0.0001). Patients receiving public health insurance made up more than half of the insurance types for CO-MRSA (56 percent); however, uTBI patients were largely from patients who were privately insured (64 percent) (p < .0001) (Table 1).

**Table 1:** Population Characteristics of Individual Level and Neighborhood Level Risks for Community-Onset Methicillin Resistant *Staphylococcus aureus* (CO-MRSA) Cases and Unintentional Traumatic Brain Injury (uTBI) Controls, 2002–2010.

Variable	Total	CO-MRSA^1^	uTBI	p-value*

	N = 39,371 N (%)	N = 4,613 N (%)	N = 34,758 N (%)	

Individual Level Characteristics				

**Race/Ethnicity**				<.0001
White (Non-Hispanic)	20,911 (53.1)	1,992 (43.2)	18,919 (54.4)	
Black (Non-Hispanic)	12,645 (32.1)	2,080 (45.1)	10,565 (30.4)	
Hispanic	3,349 (8.5)	317 (6.9)	3,032 (8.7)	
Other^2^	2,466 (6.3)	224 (4.9)	2,242 (6.5)	
**Gender**				<.0001
Male	23,651 (60.1)	2,248 (48.7)	21,403 (61.6)	
Female	15,720 (39.9)	2,365 (51.3)	13,355 (38.4)	
**Age Group, N (%)**				<.0001
>12 years	4,410 (11.2)	744 (16.1)	3,666 (10.6)	
4–12 years	14,761 (37.5)	1,317 (28.6)	13,444 (38.7)	
0–3 years	20,200 (51.3)	2,552 (55.3)	17,648 (50.8)	
**Age (years)**				
Mean (SD)	5.06 (4.80)	5.27 (5.46)	5.03 (4.71)	
**Health Insurance**				<.0001
Private	24,193 (61.5)	1,882 (40.8)	22,311 (64.2)	
Public	13,352 (33.9)	2,564 (55.6)	10,788 (31.0)	
Self Pay	1,826 (4.6)	167 (3.6)	1,659 (4.8)	
**Neighborhood Level Characteristics**				

**Household Crowding**^3^				<.0001
<=1 person/room	23,012 (58.4)	2,243 (48.6)	20,769 (59.8)	
>1 person/room	16,359 (41.6)	2,370 (51.4)	13,989 (40.2)	
**Black Population**				<.0001
<Georgia Average	28,431 (72.2)	2,821 (61.2)	25,610 (73.7)	
>= Georgia Average	10,940 (27.8)	1,792 (38.8)	9,148 (26.3)	
**Below Poverty (%)**, Mean (SD)^4^	13.90 (13.35)	17.98 (15.16)	13.36 (12.99)	<.0001

^1^ Community-Onset Methicillin-Resistant *Staphylococcus aureus* (CO-MRSA). ^2^ Other: Other, American Indian/Alaskan, Asian, Multiracial. ^3^ Measured as percentage of more than 1 occupant per room. ^4^ Measured as percentage of households with poverty to income ratio below 1. * p-value based from Chi-square tests comparing outcome by categorical variables or p-value independent samples t-test for continuous variable.

### Individual Level

Adjusted odds of risks for CO-MRSA infections are presented in Table 2. When compared to whites, blacks had 1.87 times (95 percent CI: 1.75, 200) higher odds for CO-MRSA infection. In the adjusted model, blacks, female, younger age, and public health insurance were significantly more at risk for CO-MRSA.

**Table 2:** Individual and Neighborhood Level Risk Factors for Community-Onset Methicillin Resistant *Staphylococcus aureus* Infections (CO-MRSA Cases) Compared to Unintentional Traumatic Brain Injury (uTBI Controls), Adjusted for Race, Gender, Age, and Type of Health Insurance.

Variable	Crude OR (95% CI)	P-value	Adjusted OR (95% CI)	P-value

Individual Level Risk Factors				

**Race/Ethnicity**				
White (Non-Hispanic)	Reference	–	Reference	–
Black (Non-Hispanic)	1.87 (1.75, 2.00)	<.0001	1.23 (1.14, 1.32)	<.0001
Hispanic	0.99 (0.88, 1.12)	.9117	0.57 (0.50, 0.65)	<.0001
Others^1^	0.95 (0.82, 1.10)	.4780	0.72 (0.62, 0.84)	<.0001
**Gender**				–
Male	Reference		Reference	
Female	1.69 (1.59, 1.79)	<.0001	1.70 (1.60, 1.81)	<.0001
**Age Group**				
>12 years	Reference	–	Reference	–
0–3 years	0.71 (0.65, 0.78)	<.0001	0.62 (0.57, 0.68)	<.0001
4–12 years	0.48 (0.44, 0.53)	<.0001	0.45 (0.41, 0.50)	<.0001
**Health Insurance**				
Private	Reference	–	Reference	–
Public	2.82 (2.64, 3.00)	<.0001	2.92 (2.71, 3.14)	<.0001
Self pay	1.19 (1.01, 1.41)	.0368	1.27 (1.07, 1.51)	.0058
**Neighborhood Level Factors**				

**Household Crowding**				
<=1 person/room	Reference	–	Reference	–
>1 person/room	1.05 (1.04, 1.06)	<.0001	1.02 (1.01, 1.03)	<.0001
**Black Population**				
<Georgia Average	Reference	–	Reference	–
>= Georgia Average	1.06 (1.05, 1.07)	<.0001	1.02 (1.01, 1.02)	<.0001
**Below Poverty (%)**^2^	1.03 (1.02, 1.03)	<.0001	1.03 (1.02, 1.04)	<.0001

^1^ Other: Other, American Indian/Alaskan, Asian, Multiracial. ^2^ Odds ratio is reported for changes of +/–10% change below poverty level.

### Neighborhood Level

More households with CO-MRSA infections (51 percent) lived in block groups with household crowding compared to those living in block groups with uTBI (40 percent), p < 0.0001. Similarly, higher rates of patients with CO-MRSA infections lived in communities, which were concentrated with blacks (39 percent) compared to uTBI communities (26 percent), p < 0.0001. Even with adjusting for race, gender, age, and type of health insurance, CO-MRSA patients were still 1.06 (95 percent CI 1.05, 1.07) times as likely to live in ‘concentrated black’ block groups and 1.05 (95 percent CI 1.04, 1.06) times as likely to live in crowded households. (Table 2).

### Multilevel Analyses

As shown in Table 3, once individual and neighborhood level factors were adjusted, significant main effects included race, gender, age, insurance, crowding, and poverty level. However, living in a ‘concentrated black’ area was no longer found to be a statistically significant risk, after controlling for other socio ecological and demographic risks and therefore was not included in the multi-level model. Blacks and individuals with public insurance still had higher odds for CO-MRSA, yet the correlation between individual risk factors were not as strong after introducing neighborhood level predictors.

**Table 3:** Multilevel Model of Individual and Neighborhood Risk Factors for Community-Onset Methicillin Resistant *Staphylococcus aureus* (CO-MRSA) Infections.

Variable	Adjusted OR (95% CI)	p-value

**Race/Ethnicity**		
White	Reference	–
Black	1.01 (1.00, 1.02)	.0166
Hispanic	0.93 (0.92, 0.94)	<.0001
Others^1^	0.97 (0.96, 0.98)	<.0001
**Gender**		
Male	Reference	–
Female	1.05 (1.05, 1.06)	<.0001
**Age Group**		
>12 years	Reference	–
0–3 years	0.95 (0.94, 0.96)	<.0001
4–12 years	0.92 (0.91, 0.93)	<.0001
**Insurance**		
Private	Reference	–
Public	1.11 (1.10, 1.11)	<.0001
Self Pay	1.01 (1.00, 1.03)	.1170
**Household Crowding**		
<=1 person/room	Reference	–
>1 person/room	1.01 (1.00, 1.02)	.0040
**Below Poverty (%)**^2^	1.01 (1.01, 1.02)	<.0001

^1^ Other: Other, American Indian/Alaskan, Asian, Multiracial. ^2^ Odds ratio is reported for changes of +/–10% change below poverty level.

### Spatial Analyses

**Figure 1:**
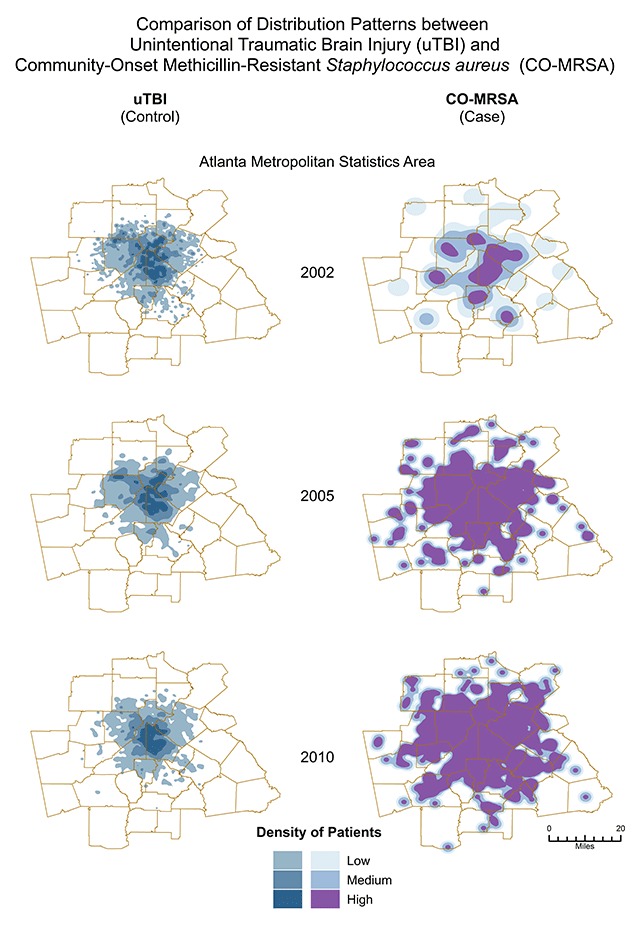
Spatially different patterns of distribution between uTBI (control) and CO-MRSA (cases.) uTBI shows a “static” pattern without much variation over the 9 years. In contrast, CO-MRSA displays patterns of spatial diffusion spread. Although spatial maps were conducted for each year, only maps reflecting spatial patterns for 2002, 2005 and 2010 are shown above.

**Figure 2:**
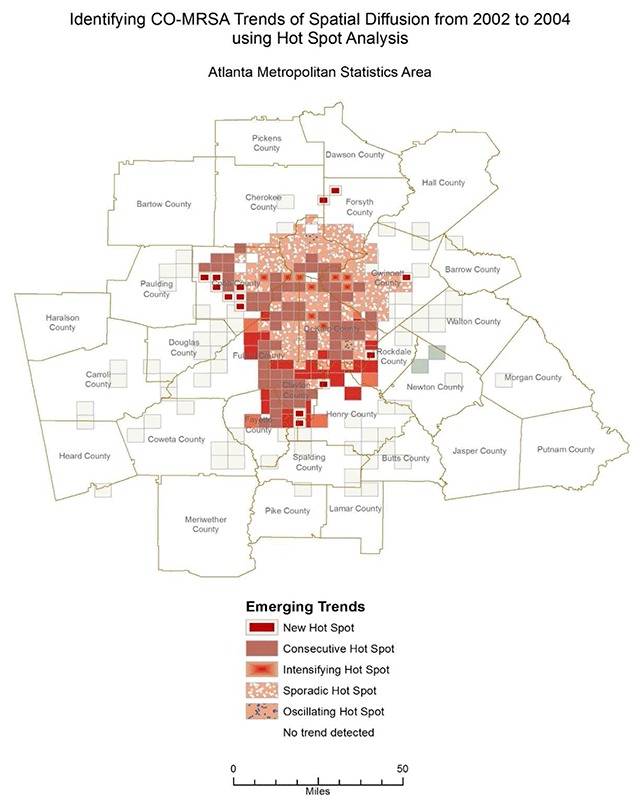
Analyzing multiple years of hot spot analysis results, Esri’s SpaceTimeCube Tool identifies emerging trends in CO-MRSA across the 20 counties of Atlanta MSA. This figure above shows statistically significant areas of new, consecutive, sporadic, and oscillating hot spots emerging and intensifying between 2002 and 2004.

**Figure 3:**
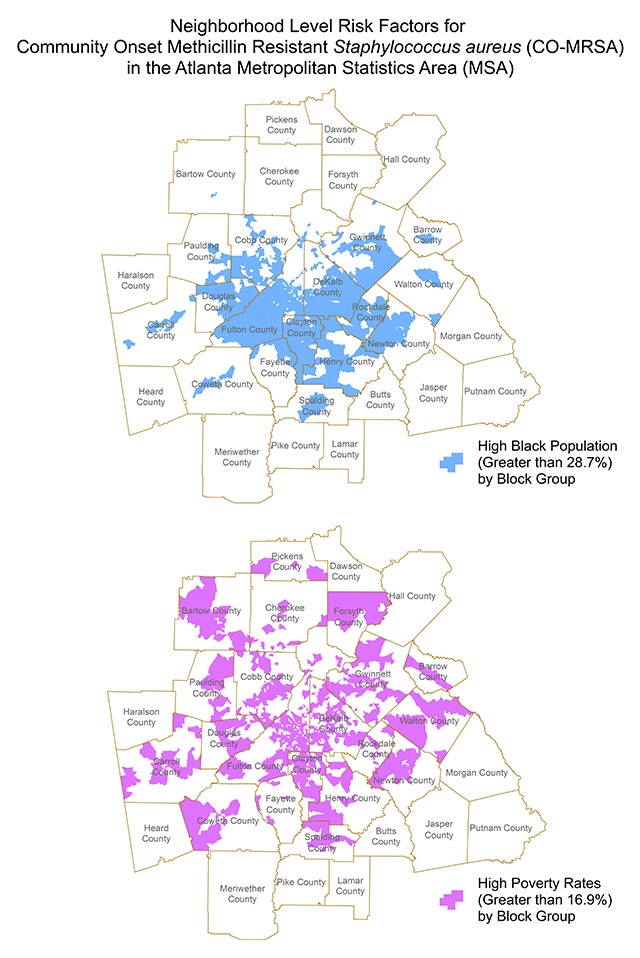
Areas of ‘concentrated black population’ are shown in the map on the left and areas of ‘high’ poverty levels are shown on the right for the period of 2002–2004. These thematic maps depict the areas which correlate with new hot spots and consecutive hots spots of CO-MRSA during the period from 2002–2004. Similar hot spot maps were created for each time period for the nine years span of 2002–2010. (Reference for poverty rate: https://www.welfareinfo.org/poverty-rate/georgia/).

The spatial distributions of MRSA and uTBI for all of the years included in the analyses indicate density patterns were statistically distinct from one another. The patterns with each progressing year were also different between CO-MRSA and uTBI: CO-MRSA patterns with each progressive year followed a spatial diffusion spread. In contrast, uTBI showed a more ‘static’ pattern without as much variation across the 9 years and high density was most apparent in and closest to the city central area of Atlanta. This was unlike CO-MRSA, which had high cluster densities in the central area but also in counties further away (Figure 1). The areas of most intense CO-MRSA were more clustered to the south of the central downtown area. CO-MRSA hot spots also had higher poverty rates, with approximately 40 percent of block groups in the highest poverty quartile. Though it appears there is more similarity between the CO-MRSA and uTBI hot spots due to the focus on the central area, the CO-MRSA hot spots mimic an expected infectious disease model originating from a central point and spreading in a uniform fashion. From the Space-Time Cube and Emerging Hot Spot analysis, we can discern different types of hotspots, which evolved over the entire time period of the study. In our example (Figure 2), we demonstrate ‘new’ hot spots for CO-MRSA over time occurred in the perimeter of the enlarging areas away from the central downtown region. Consecutive hot spots, areas where single uninterrupted run of statistically significant hot spots occurs over multiple years, ‘cropped’ up from central areas leading to the perimeter with the highest concentration occurring in counties (Clayton and Fulton counties) which are thematically considered ‘high concentrated black populations’ and impoverished communities (Figure 3). In contrast, ‘sporadic hot spots’, areas where there is an ‘on-again then off-again hot spot’, occurred in the northern part of the metropolitan area (North Fulton, Forsyth and Gwinnett counties); these counties have a smaller proportion of ‘concentrated black populations’.

## Discussion

A retrospective study was undertaken to look at individual and neighborhood level associations that highlighted neighborhoods with elevated risks of CO-MRSA from 2002–2010. A comparison was made to the non-infectious condition of unintentional TBI to purposefully identify those geographic locales, which were distinct between cases and controls. This comparison, spatially, provided further evidence of place-based risks tied with CO-MRSA cases, which are not seen with a noninfectious control (unintentional TBI). We detected areas of CO-MRSA that showed significant spatial clustering within this time period. Spatial distributions have been known to provide etiological insights into the risk of disease [2,44,45], and correlate with neighborhood conditions such as high population density [46]. The pattern of distribution indicates that CO-MRSA cases occurred in areas, which were geospatially different than the control group. Although we found risk of CO-MRSA occurred in neighborhoods with household crowding, higher concentration of blacks, and higher poverty levels, only crowding and higher poverty remained statistically significant after adjusting for other risks, compared to the control population of uTBI. In our previous study [47], we found, when compared to non-antibiotic resistant *Staphylococcus aureus* patients, CO-MRSA risk remained higher in ‘black concentrated’ communities in the risk adjusted multilevel model. This finding suggests risks associated with uTBI may be multifactorial in nature and that the reason for race/ethnic differences may be more complex between our CO-MRSA cases and controls. There are other factors using uTBI as our non-infectious control we can consider in the future, such as severity and cause of uTBI as well as including other socio ecological variables not included in this study. We plan to use this study’s findings to explore additional socio ecological factors and also to compare non antibiotic resistant *Staphylococcus aureus* to uTBI. While others have shown that lower socioeconomic status, prior medical history of chronic illness, and impaired immune response have been noted as possible reasons for differences in CO-MRSA incidence rates between race and ethnicities [7,11], these studies have not looked at the spatial relationship associated with these risks. Unlike these studies, our study addresses an individual’s place based or neighborhood characteristics’ potential role for explaining race/ethnic differences seen among CO-MRSA infected populations.

Racial disparities appear to exist within both adult and pediatric populations who develop CO-MRSA infections. Although there is data to suggest white children have high rates of community-associated MRSA skin and soft tissue infections compared to black children [38], black children overall are at increased risk for community-associated MRSA infections including those associated with skin and soft tissues [7,48,49]. This study is the first study to begin to look at both individual and area level factors, which consider socio economic (poverty rates for the area) and environmental factors (household crowding) which are location based and explore trends of CO-MRSA disease clusters over a period of time. Although risks for CO-MRSA did not include communities with black concentrated neighborhoods in our multi-level model, other variables of place-based socio economic and environmental conditions were found to be associated. Collectively, these results suggests that differences in socio economic and environmental risks among blacks that contribute to CO-MRSA may be due to conditions that are not specific to race alone. For example, household crowding factors at the household unit level may be driven by risks associated with poverty, of which race is not the only contributor. We found the racial disparity that persisted after considering all individual and area factors was between Hispanics and whites or Hispanics and blacks. This finding of Hispanic patients having a lower risk for CO-MRSA infection has been shown in other studies [39]. Moreover, Hispanics have been found to have better or similar health outcomes than whites regardless of a lower economic status [50]. Cultural factors which may contribute to our findings and account for this ‘Hispanic paradox’ [51], were not explored in this study. For example, the prevalence of breast feeding among Hispanics compared to blacks may serve a ‘protective’ role in *Staphylococcus aureus* infections [52,53]. Other cultural factors which may contribute to lower rates of CO-MRSA infections among Hispanic children are related to access to care and cultural tendency to seek health care after home or natural therapies fail to work [51].

Type of health insurance also appears to be an important predictor for risk of CO-MRSA infection. Patients without insurance (self-pay) and those with public insurance had higher odds of infection when compared to private insurance. One explanation may be that children without insurance do not seek care for these types of skin and soft tissue infections at the same rate as insured children. It is also possible that parents without insurance may seek care elsewhere, especially in low cost clinics. Children on public insurance were also more likely to present with CO-MRSA infections to the hospital or ED, compared to those who were privately insured. This finding may be attributed to the fact that access to primary care may be more challenging for these children who have public insurance and consequently, these patients, like those without any form of insurance, may not seek care until infections are more severe [52].

Crowding at the neighborhood level did have an association with CO-MRSA after adjusting for other social determinants of health, compared to non-infectious uTBI. Crowding could also be defined as population density within a block group, as infection can occur outside of the household setting but within the boundaries of a ‘community’ or ‘neighborhood’. For example, CO-MRSA infections are more likely to occur among day care attendees. Daycare centers, whether in home or at facilities, meet the definition of household crowding. Close physical proximity among people in a group setting increases the risks of touch or contact between persons, and hence, increases the probability of transferring or sharing of bacteria which reside on the skin. For CO-MRSA infections to occur, carriage of the bacterium usually precedes the actual infection. Crowding as a risk for uTBI may be likely related to the fact that higher populated areas may lead to more frequent situations which serve as the cause of uTBI, e.g., motor vehicular accidents, accidental falls, etc. It is possible if we chose another type of control group, the contribution of crowding to risk for CO-MRSA may be more evident and persist after adjusting for individual and area level variables.

Odds of having a CO-MRSA infection increased with age. This is in agreement with past research that also found older children to be more likely associated with MRSA infections [53]. The burden of colonization by CO-MRSA may increase with age, given factors which promote carriage, e.g., pubertal hormonal changes, more areas on the body where host environment (moist, not well aerated areas, etc.) is supportive of *S. aureus* carriage.

### Limitations

Data were collected from two major pediatric hospitals. A third children’s hospital which is historically affiliated with an urban public hospital was not included due to incomplete data available and so it is likely that not all cases or controls were captured for the MSA even though Children’s Healthcare of Atlanta, the largest pediatric health care system for the state of Georgia, represents the majority of all pediatric hospital discharges (~88.6 percent) during our study period [31]. We used the 20-county MSA to approximate the hospital catchment area. We did not stratify our cases based on clinical types of infections and history of previous MRSA infections, but these factors may contribute or affect significantly the individual risks for disease. Our definition of CO-MRSA did not take into consideration hospitalization history or other health care associated risks [54,55], and thus, may not be generalizable to prior research findings where these were factored.

Due to the wide geographic spread and high prevalence of CO-MRSA colonization, communities or neighborhoods are essentially reservoirs of CO-MRSA [56]. Characterization of CO-MRSA risk factors is important so that policies and strategies to reduce transmission can be developed. This is critical in the effort to prevent primary infections from occurring in the first place. Such methods often require targeting groups of individuals most at risk for infection. Hot spot analysis is a useful tool to identify communities where public health professionals should intensify control and prevention measures. For CO-MRSA, these measures often include standard hygiene practices, early identification of infections, treatment, and screening [57,58]. As antibiotic resistant bacteria prevalence continues to rise, it remains of high importance to advocate for the appropriate and correct usage of antibiotics at the community level.

In conclusion, we used a multilevel approach to describe the relationship between individual and neighborhood factors on CO-MRSA infection in children within a large urban metropolitan region in the southeastern United States. Characteristics associated with lower socioeconomic status (crowded households, public health insurance, and poverty) increased odds of CO-MRSA infection. Comparing both levels of data along with the identification of hot spots provides a more robust and integrated approach of finding and confirming factors associated with this antibiotic resistant infection.
